# Potential Herbicidal and Insecticidal (Beneficial) Properties of Nepalese Lemongrass Essential Oil

**DOI:** 10.1002/cbdv.202501095

**Published:** 2025-06-07

**Authors:** Beáta Baranová, Barbora Kudláčková, Ram Prasad Baral, Lenka Svojanovská, Qaiser Javed, Giuseppe Amato, Lucia Caputo, Laura De Martino, Rosaria Francolino, Saroj Kumar Chaudhary, Hazem S. Elshafie, Achyut Adhikari, Vincenzo De Feo, Daniela Gruľová

**Affiliations:** ^1^ Department of Ecology, Faculty of Humanities and Natural Sciences University of Prešov Prešov Slovakia; ^2^ Department of Fluid Phase Separations Institute of Analytical Chemistry of the Czech Academy of Sciences Brno Czech Republic; ^3^ Central Department of Chemistry Tribhuvan University Nepal; ^4^ Department of Pharmacy University of Salerno Fisciano Italy; ^5^ Department of Agriculture, Forestry, Food and Environmental Sciences University of Basilicata Potenza Italy

**Keywords:** antioxidant, biological activity, chemical composition, *Cymbopogon citratus* (DC.) Stapf, environmental safety, essential oil, GC/MS, herbicidal, hydrodistillation, pesticidal activity

## Abstract

*Cymbopogon citratus* (DC.) Stapf, commonly known as lemongrass, is a member of the Poaceae family and is native to tropical and subtropical regions, including South Asia. The species is renowned for its diverse applications in culinary arts, perfumery, and traditional medicinal practices. This study aimed to characterize the essential oil (EO) composition of lemongrass and evaluate its biological properties. The EO was extracted *via* hydrodistillation using a Clevenger apparatus, and its chemical profile was analyzed by gas chromatography–mass spectrometry (GC/MS). The major constituents identified were neral (35.5 ± 0.6%) and geranial (36.0 ± 0.4%), which together constitute the primary isomers of citral. The antioxidant activity, assessed using DPPH, FRAP, and ABTS assays, was moderate compared to the Trolox standard. Similarly, the EO exhibited weak tyrosinase inhibition activity. However, the EO exhibited significant herbicidal and insecticidal effects with no observed ecotoxicological risks. These findings highlight lemongrass EO as a promising eco‐friendly alternative to synthetic pesticides.

## Graphical abstract

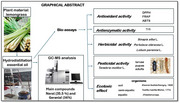

## 1 Introduction

Lemongrass, *Cymbopogon citratus* (DC.) Stapf, is a prominent member of the Poaceae family, which encompasses approximately 500 genera and around 8000 plant species [1]. The *Cymbopogon* genus comprises about 140 species that thrive in semi‐temperate and tropical regions across Asia, America, and Africa. Only a few species of *Cymbopogon* are found in Australia and Europe. Also known as “Squinant” in English, lemongrass is referred to by various colloquial names throughout the world [2]. The members of the *Cymbopogo*n genus produce volatile oils and, thus, are also known as aromatic grasses: their essential oils (EOs) are rich in monoterpenes, which include compounds such as citral, citronellal, citronellol, linalool, elemol, 1,8‐cineole, limonene, geraniol, β‐caryophyllene, methyl heptenone, and geranyl acetate as well as geranyl formate [3].

The EO of *C. citratus*, also known as citronella oil, exhibits variations in its chemical composition depending on the geographical origin. Various compounds have been identified, including terpene hydrocarbons, alcohols, ketones, esters, and primarily aldehydes. Citral, which is responsible for the strong lemon fragrance, is the main component of the EO; when two stereoisomeric monoterpene aldehydes combine to form citral, the *trans* isomer geranial predominates over the *cis* isomer neral [4]. In addition to these variations, a range of other chemical compounds can also be consistently detected, such as saponins, flavonoids, and alkaloid phenols [5]. These compounds not only contribute to the characteristic aroma of the oil but also to its potential therapeutic and biological properties [4]. Many of these volatile compounds are classified as secondary metabolites, which are thought to play significant ecological roles in plants. They facilitate interactions between plants and microorganisms, herbivorous pests, and even other plant species. As a result, plants have developed a diverse array of chemical signals that enable complex communication among several organisms. This intricate signaling system allows plants to respond to their environment and interact with other species effectively [6].

Moreover, lemongrass oil has been historically employed to treat a variety of medical conditions, as it contains a high spectrum of secondary metabolites [2]. This herbal medicine is recognized by the World Health Organization (WHO) as a significant component of healthcare, with over two‐thirds of individuals in developing nations relying on it for their health needs [7]. Traditionally, it has been used to alleviate conditions such as fever, cough, elephantiasis, flu, leprosy, malaria, and digestive disorders, including colitis, indigestion, and gastroenteritis. It also exhibits a variety of beneficial properties, including antibacterial, antifungal, analgesic, antiseptic, astringent, and antidepressant effects. Notably, lemongrass demonstrates strong antimicrobial activity against methicillin‐resistant *Staphylococcus aureus* and can serve both as an antibiotic and antiseptic, making it effective in treating conditions such as ringworm and athlete's foot [8].

Its relevance in Ayurveda persists today, because of its therapeutic properties [2]. In the literature, the bioactivities of lemongrass have been extensively studied, including antioxidant [9], antityrosinase [10], insecticidal [11], larvicidal [12], insect repellent [13], and herbicidal properties [14].

The search for sustainable alternatives to traditional pesticides (herbicides, insecticides, and so on) is increasing due to the banning of many synthetic chemicals that pose risks to human health and the environment. Concerns about pests developing resistance to these pesticides have also contributed to this shift. The EU aims for a 50% reduction in pesticide use by 2030, but from 2011 to 2022, overall pesticide sales remained stable: some countries, such as Italy and Portugal, saw a 40% decrease, while others, like Austria and Latvia, experienced nearly an 80% increase, highlighting the challenges of effective pest management without chemical solutions [6].

So, considering that (1) EOs are good candidates for natural biopesticides (herbicidal and insecticidal) due to their strong biological activities against plant pests [6], their minimal impact on non‐target organisms, thier long‐lasting effects, and their easy biodegradability into nontoxic compounds; (2) tyrosinase is a copper enzyme, that plays a key role in normal insect development [15]; the aims of this research are: (i) to study the chemical composition of the EO of *C. citratus* distilled from plants grown in Nepal; (ii) to assess its potential biological activities including, herbicidal and insecticidal effects; (iii) to hypothesize a possible mechanism of action for this EO also evaluating its antioxidant and antityrosinase capabilities.

## 2 Results

### 2.1 Identification and Quantification of the EO Composition

The EO of lemongrass was analyzed by GC/MS to determine both the major and minor compounds (Table 1). A total of 26 compounds were identified, accounting for 99.2% of the oil. The dominant compounds were neral (35.5 ± 0.6%) and geranial (36.0 ± 0.4%), present in nearly equal amounts. Geraniol was the next most abundant compound, found at a concentration of 7.5 ± 0.0%. Nine additional compounds were present in concentrations ranging from 1.0% to 3.3%, while the remaining identified components were present at levels below 1.0%.

**TABLE 1:** The composition of lemongrass essential oil determined by GC/MS.

	RI Adams	RI calculated	Compound	% ± SD
1.	921	926	Tricyclene	0.3 ± 0.0
2.	932	937	α‐Pinene	0.2 ± 0.0
3.	946	954	Camphene	0.3 ± 0.0
4.	981	984	6‐Methyl‐5‐hepten‐2‐one	1.8 ± 0.0
5.	988	989	Myrcene	1.9 ± 0.0
6.	1020	1028	*p*‐Cymene	0.1 ± 0.0
7.	1024	1033	Limonene	0.2 ± 0.0
8.	1032	1036	*cis‐*Ocimene	0.2 ± 0.0
9.	1044	1047	*trans*‐Ocimene	0.2 ± 0.0
10.	1095	1100	Linalool	1.3 ± 0.0
11.	—	1144	*trans*‐Chrysanthenol	0.2 ± 0.0
12.	1148	1154	Citronellal	1.7 ± 0.1
13.	1160	1162	Isoneral	0.9 ± 0.0
14.	1177	1181	Isogeranial	1.6 ± 0.0
15.	1223	1227	Citronellol	1.0 ± 0.0
16.	1235	1241	Neral	35.5 ± 0.6
17.	1249	1250	Geraniol	7.5 ± 0.0
18.	1264	1270	Geranial	36.0 ± 0.4
19.	1284	1291	Bornyl acetate	0.2 ± 0.0
20.	1379	1377	Geranyl acetate	0.9 ± 0.0
21.	1389	1398	β‐Elemene	0.2 ± 0.0
22.	1417	1434	β‐Caryophyllene	1.7 ± 0.0
23.	1432	1440	*trans*‐α‐Bergamotene	0.2 ± 0.0
24.	1452	1471	α‐Humulene	0.2 ± 0.0
25.	1522	1525	γ‐Cadinene	3.3 ± 0.1
26.	1582	1599	Caryophyllene oxide	1.0 ± 0.0
			Total	99.2 ± 0.0

*Note*: Bold labeled compounds were identified by comparison with the spectra of authentic standards. Results are reported as the mean ± SD of three experiments.

Abbreviations: RI Adams, retention index from literature—Adams; RI calculated, retention index determined with respect to homologous series of *n*‐alkanes (C_9_–C_23_) on a DB‐5 column.

### 2.2 Antioxidant Activity

Recognizing that a single antioxidant assay provides only partial information about sample antioxidant properties, this study employed three different methods to obtain more comprehensive data. Table 2 reported the antioxidant activity of *C. citratus* EO measured by the DPPH, ferric reducing antioxidant power (FRAP), and ABTS assays. The results indicated that the EO exhibited a higher radical‐scavenging capacity with the DPPH radical compared to the ABTS radical cation, while the FRAP value of the EO was found to be low.

**TABLE 2:** Antioxidant activity of *Cymbopogon citratus* EO.

	EO *C. citratus*		Trolox
DPPH (IC_50_ mg/mL)	5.13^b^ ± 0.13	DPPH (IC_50_ µg/mL)	3.72^a^ ± 0.08
FRAP (mmol Fe^2+^ eq/g)	3.28^b^ ± 0.19	FRAP (mmol Fe^2+^ eq/g)	9.48^a^ ± 0.34
ABTS (TE mg/g)	1.31 ± 0.02	ABTS (TE mg/g)	—

*Note*: Results are reported as the mean ± SD of three experiments. Trolox was used as the reference standard. Letters in the same graphic indicate that are significantly different at *p* < 0.05, according to a one‐way ANOVA followed by Tukey's post hoc test.

### 2.3 Anti‐Tyrosinase Activity

Table 3 reports the anti‐tyrosinase activity of the EO. The sample demonstrated greater inhibitory activity against the enzyme in the diphenol reaction compared to the monophenolase reaction. However, the activity of the EO was lower than that of kojic acid, which served as the standard reference.

**TABLE 3:** Anti‐tyrosinase activity of *Cymbopogon citratus* EO.

	EO of *C. citratus* (IC_50_ mg/mL)	Kojic acid (IC_50_ µg/mL)
Tyrosinase (IC_50_, tyrosine monophenolase reaction)	20.50^b^ ± 0.22	12.40^a^ ± 2.24
Tyrosinase (IC_50_, L‐DOPA diphenolase reaction)	15.30^b^ ± 0.18	70.70^a^ ± 3.04

*Note*: Results are reported as the means ± SD of three experiments. Letters in the same graphic indicate that are significantly different at *p* < 0.05, according to a one‐way ANOVA followed by Tukey's post hoc test.

### 2.4 Kinetic Study

Given the inhibitory activity against the tyrosinase enzyme, a kinetic study was conducted to investigate the type of enzymatic inhibition and determine its maximum speed (*V*_max_) and *K*_m_. *K*_m_ represents the substrate concentration at which the reaction rate is equal to half of the maximum velocity, while *V*_max_ is the maximum velocity at which an enzyme can catalyze the reaction, occurring when all enzyme active sites present are completely saturated with substrate. These data are represented in the Tables 4 and 5.

**TABLE 4:** *V*_max_, *K*_m_, values for tyrosinase (monophenolase reaction) inhibition by *Cymbopogon citratus* EO, geranial, and citral.

	Tyrosinase (monophenolase reaction)		
	*V*_max_ (µM/min)	*K*_m_ (mM)	Type of inhibition
*C. citratus* EO	6.93	2.75	Non‐competitive
Geranial	1.76	1.5	Uncompetitive
Citral	7.4	2.82	Non‐competitive

Abbreviations: *K*_m_, substrate concentration where the corresponding reaction rate is ½ of *V*_max_; *V*_max_, maximum rate of enzymatic reaction.

**TABLE 5:** *V*_max_, *K*_m_, values for tyrosinase (diphenolase reaction) inhibition by *Cymbopogon citratus* EO, geranial, and citral.

	Tyrosinase (diphenolase reaction)		
	*V*_max_ (µM/min)	*K*_m_ (mM)	Type of inhibition
*C. citratus* EO	26.58	2.53	Non‐competitive
Geranial	n.d	n.d	n.d
Citral	n.d	n.d	n.d

Abbreviations: *K*_m_, substrate concentration where the corresponding reaction rate is ½ of *V*_max_; n.d., not determined; *V*_max_, maximum rate of enzymatic reaction.

**FIGURE 1:**
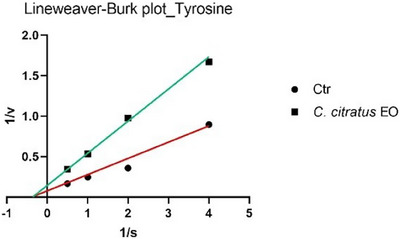
Lineweaver–Burk plots of tyrosinase inhibition (monophenolase reaction) in the absence (CTR) or presence of *Cymbopogon citratus* EO.

**FIGURE 2:**
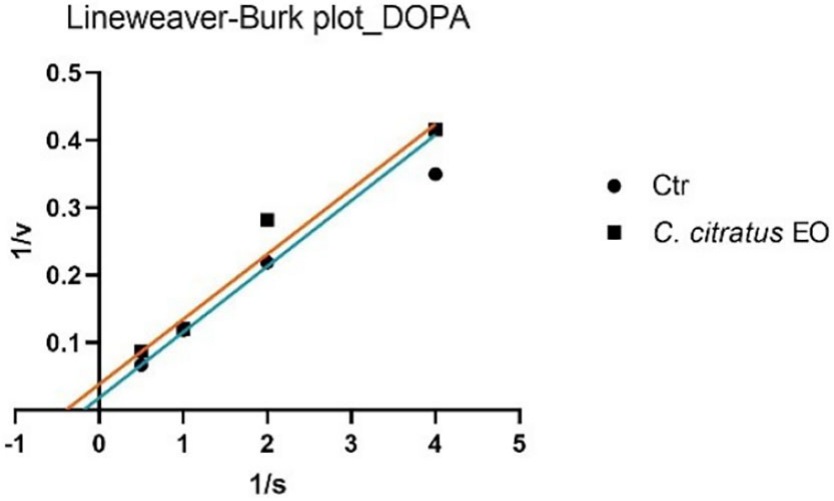
Lineweaver–Burk plots of tyrosinase inhibition (diphenolase reaction) in the absence (CTR) or presence of *Cymbopogon citratus* EO.

The analysis of the Lineweaver–Burk plots (Figures 1 and 2) revealed that the EO of *C. citratus* inhibits tyrosinase through different mechanisms, depending on the type of reaction. Specifically, EO acts as a non‐competitive inhibitor in the monophenolase reaction, while it exhibits uncompetitive inhibition in the diphenolase reaction.

**FIGURE 3:**
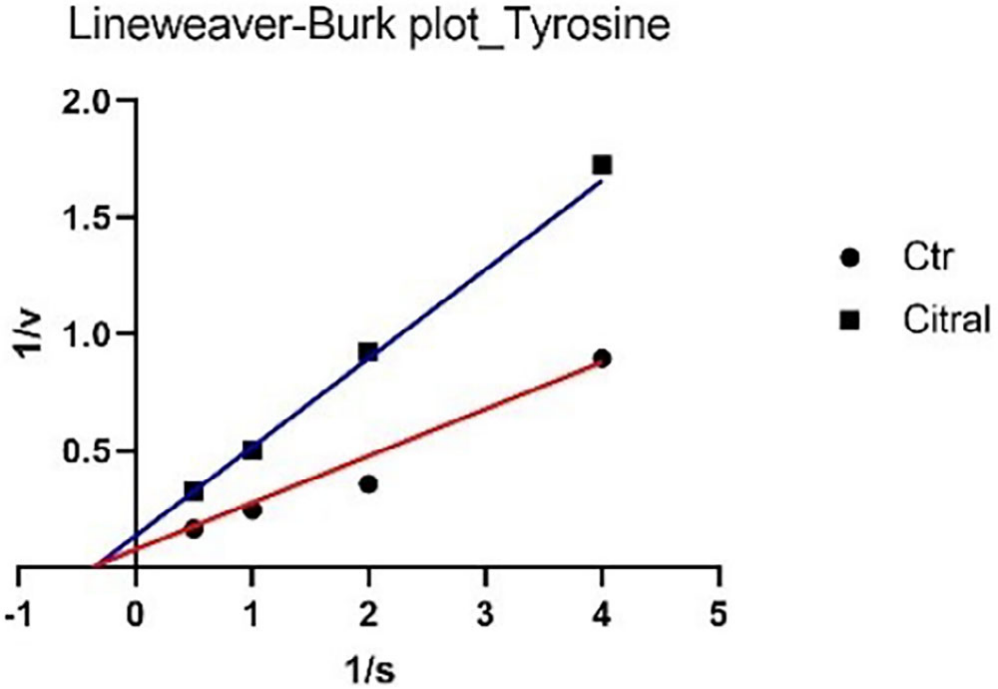
Lineweaver–Burk plots of tyrosinase inhibition (monophenolase reaction) in the absence (CTR) or presence of citral.

**FIGURE 4:**
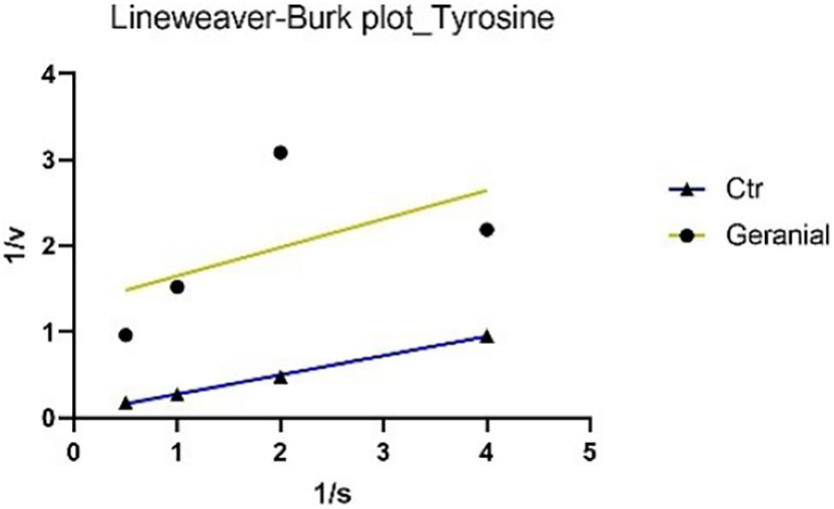
Lineweaver–Burk plots of tyrosinase inhibition (monophenolase reaction) in the absence (CTR) or presence of geranial.

The analysis of the main constituents revealed that citral and geranial (Figures 3 and 4) are active exclusively in the monophenolase reaction, but they employ distinct inhibitory mechanisms: citral demonstrates non‐competitive inhibition (binding to the enzyme independent of the substrate, lowering *V*_max_ while leaving *K*_m_ unchanged), while geranial acts as an uncompetitive inhibitor (binding only to the enzyme‐substrate complex, reducing both *V*_max_ and *K*_m_).

### 2.5 Herbicidal Activity

The herbicidal activity of lemongrass EO was tested on seeds of three plant species that are commonly considered weeds in European agrosystems. A significant reduction in germination was observed only at the highest EO concentration tested (625.00 µg/mL): at this concentration, the germination rates of *Lolium perenne*, *Portulaca oleracea*, and *Sinapis alba* were significantly reduced compared to the control (Tables 6 and 7). For each model organism, a significant negative linear relationship was found between EO concentration and germination rate.

**TABLE 6:** Percentage (%) of seeds that germinated following the application of four concentrations of lemongrass EO.

Germination (%)		Control	78.12 µg/ mL	156.25 µg/mL	312.50 µg/mL	625.00 µg/mL
*Lolium perenne*	Min/max	60/100	50/90	40/90	60/90	0/90
	Mean ± SD	73.33 ± 12.25	74.44 ± 13.33	74.44 ± 17.40	73.33 ± 11.18	47.78 ± 30.32^*^
*Portulaca oleracea*	Min/max	80/100	70/100	70/100	60/100	0/60
	Mean ± SD	90.00 ± 7.07	91.11 ± 10.54	86.67 ± 11.18	81.11 ± 11.67	16.67 ± 22.36^***^
*Sinapis alba*	Min/max	60/90	60/90	60/100	60/100	0/60
	Mean ± SD	76.67 ± 12.25	78.89 ± 12.69	81.11 ± 16.91	71.11 ± 12.69	35.56 ± 17.40^***^

*Note*: Findings from the one‐way ANOVA test assessing the significance of differences in seed germination between treatments and controls are indicated as follows:

* *p* ≤ 0.05;

*** *p* ≤ 0.001.

**TABLE 7:** Lemongrass EO 50% inhibitory concentration (IC_50_).

		95% Fiducial CI	
	IC_50_ µg/mL	Lower µg/mL	Upper µg/mL
*Lolium perenne*	612.07	393.26	981.79
*Portulaca oleracea*	440.65	324.74	598.38
*Sinapis alba*	905.81	464.13	1832.19

The root elongation of *P. oleracea* was the most strongly inhibited among the model organisms used, as the root length of purslane was significantly lower compared to the control group at all four EO concentrations (Table 8). Furthermore, a significant negative correlation was observed between EO concentration and root growth for each model organism studied.

**TABLE 8:** Roots length (cm) after the application of four concentrations of lemongrass EO.

Root length (cm)		Control	78.12 µg/mL	156.25 µg/mL	312.50 µg/mL	625.00 µg/mL
*Lolium perenne*	Min/max	1.39/2.00	1.43/3.13	1.18/2.09	0.93/1.76^***^	0.53/1.30^***^
	Mean ± SD	1.72 ± 0.22	2.03 ± 0.52	1.63 ± 0.29	1.20 ± 0.31	0.89 ± 0.29
*Portulaca oleracea*	Min/max	1.76/2.35	1.36/2.06	1.33/2.32	0.70/1.86	0.80/1.10
	Mean ± SD	2.08 ± 0.22	1.63 ± 0.22^***^	1.74 ± 0.32^*^	1.33 ± 0.36^***^	0.93 ± 1.28^***^
*Sinapis alba*	Min/max	1.75/5.38	3.37/6.23	1.91/4.27	1.11/3.80	0.53/2.10
	Mean ± SD	2.86 ± 1.09	4.35 ± 0.95^**^	3.18 ± 0.84	2.18 ± 0.91	1.16 ± 0.59^**^

*Note*: Findings from the one‐way ANOVA test assessing the significance of differences in root length between treatments and controls are indicated as follows:

* *p* ≤ 0.05;

** *p* ≤ 0.01;

*** *p* ≤ 0.001.

### 2.6 Ecotoxicity Test

No mortality was observed in *Tubifex* at any of the EO concentrations tested in the herbicidal assay, even after extended exposure for 24 h. The same result was found for the soil redworm, *Eisenia foetida*. In contrast, the most sensitive organism to lemongrass EO was the aquatic larva of *Chaoborus* spp.: at the highest doses of the oil (625.00 and 312.50 µg/mL), a mortality rate of 25.5% (±7.09) and 5.5% (±2.42), respectively, was observed.

### 2.7 Insecticidal Test

After 24 h from the application, the first death specimens and signs of necrosis were noticed. In the control group, mortality across all developmental stages was less than 1%. The results indicate that mortality rates varied among the developmental stages of the yellow mealworm: at a dose of 0.889 µg/insect, the average mortality of larvae was 24.07%, while the mortality rates for pupae and adults were significantly higher at 74.07% and 87.04%, respectively (*p* < 0.001, in comparison to larvae mortality).

Similarly, the LD_50_ for larvae was calculated as 11.13 µg/insect (95% CI: 3.44–36.01), while the LD_90_ was 207.76 µg/insect (95% CI: 64.19–672.31); for pupae the LD_50_ was 0.76 µg/insect (95% CI: 0.56–1.02) and LD_90_ was 1.77 µg/insect (95% CI: 1.29–2.44); for adults, the LD_50_ was 0.67 µg/insect (95% CI: 0.55–0.82) and LD_90_ was 1.16 µg/insect (95% CI: 0.94–1.42) consequently. However, no statistically significant differences were found in the LD_50_ or LD_90_ values among the developmental stages.

A significant negative linear correlation (*p* < 0.001) was observed between lemongrass EO concentration and mortality in *T. molitor* larvae, pupae, and adults.

## 3 Discussion

### 3.1 Identification and Quantification of the EO Composition

The composition of lemongrass EOs varies according to several factors, including geographical origin, plant variety, the plant part used, the method of drying or extraction, and environmental and agronomic conditions. The main constituent of lemongrass EOs is citral, which consists of two geometric isomers: geranial (*E*‐isomer; *trans*‐citral; α‐citral; or citral A) and neral (*Z*‐isomer; *cis*‐citral; *β*‐citral; or citral B). The content of this isomeric mixture can be used as a quality marker for lemongrass EO. *C. citratus* EO should contain at least 75% of citral to be considered a high‐quality product; in the oil reported here, 72% of citral was found. In addition to citral, myrcene, geraniol, citronellol, and limonene are also present in significant amounts [9, 16, 17]. Majewska et al. [9] reported that the significant variations in myrcene content (which ranged from 0.80% to 25.37%) found in the EOs of lemongrass are directly related to the geographical regions around the world.

According to Akhila et al. [16], the content of EOs from *C. citratus* ranged between 10% and 48% for geranial, 3% and 43% for neral, and 2.6% and 40% for geraniol, which corresponds to the presented data. Similar results were previously reported [9], with geranial (0.99%–48.14%) dominating over neral (0%–38.32%) and geraniol (1.34%–21.86%). Kiełtyka‐Dadasiewicz et al. [17] also found geranial (24.9%–48%), neral (10.5%–35.1%), and geraniol (0.4%–6.6%).

The results obtained were in good agreement with those published by Dangol et al. [18], who reported a comparable content of neral (36.1%) but a higher level of geranial (53.1%) and a lower amount of geraniol (0.4%) in EOs from Eastern Nepal *C. citratus* leaves. Notably, myrcene was not detected in these samples.

### 3.2 Antioxidant Activity

Moderate antioxidant activity was observed in *C. citratus* EO, with a greater effect on radical inhibition than on ferric‐reducing power. The antioxidant activity of *C. citratus* EO was studied by many researchers but the results were controversial. For instance, Yen and Lin [19] reported a very high DPPH IC_50_ > 30 mg/mL, and Li et al. [20] showed an antioxidant capacity of *C. citratus* EO weaker than that of Vitamin C. In contrast, *C. citratus* EO sample studied by Salaria et al. [21] exhibited significant antioxidant activity across all methods used with IC_50_ values of 47.53 µg/mL for DPPH, 30.7 µM for FRAP, and 27.87 µg/ml for ABTS, respectively.

The discrepancies in these results are likely due to differences in plant origin, extraction methods, and the specific antioxidant assays used [9]. In the literature, Habib and collaborators [22] reported the IC_50_ of 6.9 µg/mL for citral, in the DPPH assay.

### 3.3 Anti‐Tyrosinase Activity

Tyrosinase plays an important role in normal insect development processes, including cuticular tanning, sclerotization, wound healing, and nodule formation for defence against foreign pathogens [15]. Consequently, inhibiting tyrosinase can disrupt these defense mechanisms or cause abnormal softening of the insect body, making it a promising target for novel insecticides [23].

In this study, the *in vitro* inhibition of tyrosinase by *C. citratus* EO was examined to evaluate its insecticidal potential. The results showed that the sample exhibited moderate inhibitory activity on the enzyme in the diphenolase reaction and low activity in the monophenolase reaction.

To date, only two studies have reported data on the potential inhibition of tyrosinase by *C. citratus* EO, and neither has linked this activity to possible insecticidal effects [24, 25]. In particular, Saeio et al. [24] reported an IC_50_ value of 0.5 mg/mL for the diphenolase reaction, while Aumeeruddy‐Elalfi et al. [25] showed an IC_50_ value of 132 µg/mL for the monophenolase reaction. In both cases, the EO appeared to be more active than the sample in this study; however, this discrepancy may be attributed to the higher substrate concentrations used in their experiments compared to those in the current assay. For citral, a main component of the oil, Capetti et al. reported a tyrosinase IC_50_ of 121.8 µg/mL [26]. Citral can inhibit the enzyme through a non‐competitive mechanism of action [27, 28]. Regarding the kinetic study, in Figures 1 and 3, the Lineweaver–Burk plots for tyrosinase show that the lines intersect to the left of the *X*‐axis and have different *Y*‐intercepts. This pattern indicates that both the EO and citral decrease the rate of the monophenolase reaction catalyzed by tyrosinase without altering the Michaelis constant *K*_m_. Therefore, this type of inhibition can be classified as non‐competitive. In Figures 2 and 4, the Lineweaver–Burk plots for tyrosinase display parallel lines, indicating that both the EO (in the diphenolase reaction) and geranial (in the monophenolase reaction) reduce as the reaction rate (*V*_max_) as the Michaelis constant *K*_m_. This pattern is characteristic of uncompetitive inhibition.

### 3.4 Herbicidal Activity

EOs and EO‐based products are considered promising candidates for biocontrol agents due to their safe, bioactive, biodegradable, ecologically, and economically viable properties [29]. Citral, a mixture of the isomers neral and geranial, has been identified in several studies as a key compound responsible for allelopathic effects [29, 30, 31].

Laboratory bioassays have shown that high concentrations of EO can significantly inhibit the germination and seedling growth of *Echinochloa crus‐galli* (L.) P. Beauv., demonstrating strong weed‐suppressing abilities; hence, these findings could be a base for developing natural herbicides [32].

In addition, other plant EOs, such as *Citrus x aurantiifolia* (Christm.) Swingle EO and its major constituents, limonene (∼41%) and citral (∼28%), were demonstrated to affect mitotic activity and induce chromosomal abnormalities in three grassy agricultural weeds—*Avena fatua* L., *E. crus‐galli*, and *Phalaris minor* Retz. Citral showed the most toxicity, followed by *C. aurantiifolia* oil [33].

The allelopathic effect of the EO from *Elionurus muticus* (Spreng.) Kuntze, collected in Brazil and rich in citral, was also evaluated. The results showed that both treatments (EO and citral) exhibited an inhibitory effect on the germination of lettuce and onion [34].

### 3.5 Ecotoxicity Test

Although lemongrass EO may present some toxicity risks to non‐target organisms, the concentrations used in phytotoxicity bioassays, effective in inhibiting germination and root growth of model plants, were not toxic to soil or semiaquatic organisms, that is, sludge worms and earthworms at all. However, a slight increase in mortality was noted in glass worms, which represent aquatic organisms, indicating that caution should be exercised if lemongrass EO is to be used in practical applications. As with conventional herbicides, it remains important to follow proper application techniques to minimize unintended impacts on non‐target species [35, 36, 37].

### 3.6 Insecticidal Test

The insecticidal activity of lemongrass EO was evaluated in a dose‐response bioassay conducted under laboratory conditions against yellow mealworm larvae, pupae, and adults. Increasing doses of lemongrass EO showed toxic effects on yellow mealworm larvae, pupae, and adults 48 h after topical application. Our results indicate that different developmental stages of *T. molitor* exhibit varying sensitivities to lemongrass EO, consistent with the findings of Plata‐Rueda et al. [38, 39]. However, in contrast to their observations, our study found that larvae were the least sensitive while adults were the most sensitive to lemongrass EO; small quantities of lemongrass EO were toxic to *T. molitor*, indicating a decrease in tolerance with age. Generally, the insecticidal effect of lemongrass EO was comparable to that of oregano EO (*Origanum vulgare* L., Lamiales: Lamiaceae) as reported by Plata‐Rueda et al. [39], who found the following results: larvae LD_50_ = 3.03 µg/insect, pupae LD_50_ = 5.01 µg/insect, adults LD_50_ = 5.12 µg/insect of *T. molitor*. These findings suggest the potential of lemongrass EO limiting the survivorship of yellow mealworm.

### 3.7 Mechanism of Action of *C. citratus* EO

The EO of *C. citratus*, primarily rich in citral (neral + geranial), limonene, geraniol, and β‐myrcene, exhibits a wide range of biological activities that can be partially explained by the known mechanisms of action of these volatile compounds. Citral, the major constituent, is recognized for its ability to disrupt cell membranes, leading to loss of membrane integrity and leakage of intracellular contents [40]. In addition, citral interferes with redox processes and may influence gene expression related to stress responses and apoptosis [41]. Several studies report that citral may inhibit key enzymes such as acetylcholinesterase (AChE), α‐amylase, and α‐glucosidase, highlighting its potential as a therapeutic agent in the managing neurodegenerative diseases and type 2 diabetes mellitus [42, 43]. The inhibition mechanism may be either competitive or non‐competitive, depending on its concentration and interaction with the enzyme active site. Geraniol and β‐myrcene also contribute significantly to the EO of *C. citratus* bioactivity. Geraniol has been shown to modulate mitochondrial function and induce apoptosis via caspase‐dependent pathways [44], while β‐myrcene is known for its anti‐inflammatory and antioxidant properties, further enhancing the overall synergistic effect [45] of *C. citratus* EO. EOs are complex mixtures of multiple components, and synergistic as well as antagonistic interactions between individual constituents may affect their exact inhibitory activity. Moreover, the *in vitro* models used in this study present limitations as they do not account for metabolism, bioavailability, or potential toxicity *in vivo*.

## 4 Conclusion

A series of bioassays were conducted to evaluate the biological activities of *C. citratus* collected in Nepal. The chemical composition was studied, with the dominant components identified as neral and geranial, which together represent citral. As mentioned in a previous study, lemongrass is a typical aromatic and medicinal plant that can be used for different purposes. Our investigation expanded the understanding of lemongrass EO by assessing its antioxidant, anti‐enzymatic, and pesticidal activities, all of which showed promising results even at lower concentrations. Notably, the ecotoxicological assay revealed no significant adverse effects on non‐target organisms, suggesting a favorable safety profile for environmental applications. Consequently, this herbaceous plant holds significant potential for various applications within the agricultural sector. Further studies are needed to determine the exact mode of action; however, the current findings regarding pesticidal activity suggest that *C. citratus* could serve as another valuable plant source for implementation in agroindustry and weed management.

## 5 Experimental Section

### 5.1 Plant Materials

The plant material utilized for this study was collected from the Surkhet District, Karnali Province, Nepal, in August 2022. Plant material was identified by Dr. Hem Raj Poudel, Botanist of National Herbarium and Plant Laboratories (NHPL), in Lalitpur, Bagmati Province, Nepal. A voucher specimen (cc‐22) is deposited in the same Department. After harvesting, the lemongrass leaves were detached from their stalks and subjected to air drying. Once dried, the plant material was segmented into pieces approximately 4–8 mm in length. Distillation procedures employed distilled water, with a standardized lemongrass mass of 200 g per batch, encompassing both leaves and stalks.

### 5.2 Extraction of EO

EO extraction was performed by hydrodistillation using a Clevenger‐type apparatus [46]. The leaves and stalks were processed under optimized conditions to yield a mixture of oil and water. Dichloromethane was employed to isolate the EO from the aqueous phase. The resultant EO was subsequently dried using anhydrous sodium sulfate and stored at temperatures between 4°C and 6°C in a dark environment for subsequent analyses.

### 5.3 GC/MS Identification of Main Compounds

Chemical profiling of the EO was achieved through GC/MS analysis using a Thermo Trace GC Ultra system integrated with a TriPlus autosampler and an ion‐trap Polaris Q mass spectrometer in electron impact (EI) mode at 70 eV. The chromatographic separation was achieved on a DB‐5 capillary column (30 m × 0.25 mm i.d., film thickness 0.25 µm). The temperature program initiated at 45°C (held for 2 min), increased to 250°C at a rate of 10°C/min, followed by a further rise to 300°C at 30°C/min, and was maintained for an additional 2 min. Helium served as the carrier gas at a flow rate of 0.8 mL/min. Samples (1 µL) dissolved in *n*‐hexane were injected in splitless mode. Mass spectra were captured in full scan mode (*m*/*z* 50–650 amu) after a solvent delay of 4.7 min. The injector and the transfer line were maintained at 225°C, and the ion source at 220°C. The data analysis was performed using Xcalibur 2.2 software. Compound identification was carried out by comparison with co‐injected pure standards, as well as by matching mass spectral fragmentation patterns and retention indices with the literature data [47] or reference libraries (NIST MS Search 2.0). The retention indices of the compounds were determined with respect to homologous series of *n*‐alkanes (C_9_–C_23_; Merck KGaA, Germany).

### 5.4 Antioxidant Activity

#### 5.4.1 DPPH Assay

The DPPH radical scavenging activity was assessed using the protocol by Xiang et al. [48]. with minor adjustments. The EO was dissolved in methanol to prepare a range of final concentrations from 1.25 to 10 mg/mL. Methanolic solutions of EO were mixed with a ^•^DPPH solution (60 µM) in cuvettes, achieving a final volume of 1 mL. Absorbance at 515 nm was measured after a 45‐min incubation using a UV spectrophotometer (Thermo Fisher Scientific). Results were expressed as IC_50_ values (sample concentration reducing ^•^DPPH absorbance by 50%) and compared to the Trolox standard.

#### 5.4.2 FRAP Assay

The FRAP assay followed the Benzie and Strain method [49] with slight modifications. Reaction mixtures, prepared in 96‐well plates, included FRAP reagent and were incubated at 37°C in the dark for 30 min. Absorbance was measured at 593 nm, and results were expressed as mmol Fe^2+^/g of extract. Trolox served as the standard reference.

#### 5.4.3 ABTS Test

The ABTS radical scavenging activity was evaluated using the method of Re et al. [50]. ABTS radical cations (ABTS^•+^) were generated by reacting ABTS with potassium persulfate and incubating the mixture in the dark for 16 h. The resulting ABTS^•+^ solution was then diluted to achieve an absorbance of 0.800 at 734 nm. EO samples, at concentrations ranging from 5 to 20 mg/mL, were mixed with ABTS^•+^ in 96‐well plates, and the absorbance at 734 nm was measured. Results were expressed as µM Trolox equivalent antioxidant capacity (TEAC) per gram of sample.

### 5.5 Tyrosinase Inhibition Assay

Tyrosinase inhibitory effect was determined by evaluating monophenolase and diphenolase activities using a modified protocol adapted from Khatib et al. [51]. Reaction mixtures were prepared in 96‐well plates by combining EO (1–30 mg/mL), tyrosinase enzyme solution, sodium acetate buffer (50 mM, pH 6.8), and the appropriate substrate (l‐DOPA or l‐tyrosine). After incubation, optical density at 492 nm was measured. The inhibitory effect was quantified as IC_50_ values (the concentration required to inhibit 50% of enzyme activity), and results were benchmarked against kojic acid, which served as the positive control.

### 5.6 Kinetic Study

Lineweaver–Burk plots were used to linearize the substrate‐velocity data and to investigate the kinetic parameters determining the type of inhibition, the Michaelis–Menten constant (*K*_m_), and maximum velocity (*V*_max_) values. In addition, the inhibition constant (*K*_i_) was determined to express the affinity of the EO for binding to enzymes [52]. For the kinetics study, the experiments were carried out as described in previous paragraphs, with the exception that several substrate concentrations were used.

The concentration of the tyrosinase was kept constant at 200 U/mL and different concentrations of tyrosine (0.5–2.5 mM) in the absence (control) and in the presence of 400 µg/mL of EO were used. The reaction was started by the addition of substrate, and monitored with UV spectrophotometer at 412 nm (Thermo Fischer Scientific, Vantaa, Finland Varian, CARY 100 Bio, USA), at 1‐min intervals over a period of 30 min. All experiments were carried out in triplicate.

### 5.7 Herbicidal Activity

The herbicidal potential of the EO was tested using the modified method of Mancini et al. [53]. Four EO doses (78.12, 156.25, 312.50, and 625.00 mg/mL) were applied to sterilized seeds of *S. alba* L. (white mustard), *P. oleracea* L. (purslane), and *L. perenne* L. (perennial ryegrass), placed on filter paper in Petri dishes. After treatment, the dishes were incubated at 22 ± 1°C with an 8:16‐h light/dark photoperiod. Germination percentages and root lengths were recorded after 120 h.

### 5.8 Ecotoxicity

The ecotoxicity of the EO was assessed using three model organisms representing soil, semi‐aquatic, and aquatic environments, each tested at the same concentrations as in the herbicidal bioassay. The soil redworms, *E. foetida* Savigny, 1826 (Annelida, Oligochaeta: Tubificidae), a standard model organism in ecotoxicological studies, were tested for 48 h using a contact filter paper test in accordance with the OECD Guideline [54], with slight modification [55]. Three independent trials were conducted, with a total of 15 worms per concentration. The semiaquatic sludge worms *Tubifex tubifex* Müller, 1774 (Annelida, Oligochaeta: Lumbricidae) were subjected to a rapid 3‐min test for acute toxicity determination [56]. If no mortality was observed after the initial 3 min, exposure was extended up to 24 h, with mortality checked at set intervals. Three independent trials were conducted, totaling 90 specimens for each concentration. The aquatic larvae of glass worms, *Chaoborus* spp. (Diptera: Chaoboridae), which are harmless, non‐biting representatives of aquatic invertebrates, were tested following the standard protocol suggested by the World Health Organization [57] with minor modifications [58]. Again, three independent trials were conducted, totaling 90 specimens for each concentration.

### 5.9 Insecticidal Activity

Yellow mealworm (*Tenebrio molitor* L., Coleoptera: Tenebrionidae) larvae, pupae, and adults were used to evaluate the insecticidal (lethal) activity of lemongrass EO via a contact‐toxicity test, following the methods of Plata‐Rueda et al. [38, 39]. The insects were obtained from a breeding stock maintained in the Department of Ecology at FHNS, University of Prešov, Slovakia. This stock culture was kept in plastic trays measuring 35 cm long × 19.5 cm wide × 11.5 cm high, at room temperature, and was fed a mixture of wheat flour, semolina, and bread crumbs, supplemented regularly with fresh vegetables. Based on preliminary fit‐range tests, three EO doses producing differential mortality were selected for the contact‐toxicity test to determine the LD_50_ and LD_90_ values. The experiment involved yellow mealworm larvae, pupae, and adults that were healthy and free of visible damage. The EO was applied on the thorax of each larva, pupa, or adult of *T. molitor*, using a micropipette. Three independent trials were conducted for each EO dose, totaling 108 specimens per dose. Bioassays were checked after 24 and 48 h, during which the specimens were evaluated for movement and signs of necrosis.

### 5.10 Data Analyses

The anti‐germinative and phytotoxic activity of lemongrass EO was used to assess the plant herbicidal potential. Anti‐germinative activity was expressed as the average percentage of germinated seeds (in %), while phytotoxic activity was determined by measuring the mean root length (in cm). Minimal (min), maximal (max), average (mean) as well as standard deviation (SD) were calculated using univariate statistics. One‐way ANOVA (OWA) was used to assess statistical significance between control and treatment groups for both germination percentage and root length, with significance thresholds set at *p* < 0.05, *p* < 0.01, and *p* < 0.001. In addition, the germination inhibition activity of lemongrass EO was evaluated. Data are presented as the 50% inhibitory concentration (IC_50_) along with their respective 95% confidence intervals (95% CIs). The data presented are the mean of three replicates, and the IC_50_ value was obtained using log‐probit analysis. Simple linear regression analysis was used to assess potential correlations between germination activity/root length, and EO dose was used, with significant correlations depicted accordingly. All statistical analyses were performed using PAST version 4.03c [59].

The crude mortality data obtained from the *T. molitor* larvicidal bioassay were corrected using Abbott's formula (1925); then, insecticidal data (48‐h mortality), were subjected to Finney's probit analysis to determine the LD_50_, LD_90_, and 95% CIs for the upper and lower confidence limits (UCL/LCL) [60]. The data presented are the mean of three replicates. Differences between developmental stages in LC_50_/LC_90_ values were determined using OWA [59].

For the antioxidant and anti‐enzymatic experiment, GraphPad Prism version 6.0 (GraphPad Software Inc., San Diego, CA, USA) for Windows was used to analyze data obtained through *t*‐test analysis, conducted three times for each determination at a significance level of *p* < 0.05.

## Author Contributions

**Daniela Gruľová**: conceptualization, writing – original draft preparation, writing – review and editing, funding acquisition, supervision. **Achyut Adhikari**: conceptualization, writing – review and editing. **Laura De Martino**: conceptualization, writing – review and editing, methodology, formal analysis and investigation, writing – original draft preparation. **Vincenzo De Feo**: conceptualization. **Beáta Baranová**: methodology, formal analysis and investigation, writing – original draft preparation. **Barbora Kudláčková**: methodology, formal analysis and investigation, writing – original draft preparation. **Lucia Caputo**: methodology, formal analysis and investigation, writing – original draft preparation. **Lenka Svojanovská**: formal analysis and investigation, writing – original draft preparation. **Qaiser Javed**: formal analysis and investigation. **Giuseppe Amato**: formal analysis and investigation, writing – original draft preparation. **Rosaria Francolino**: formal analysis and investigation. **Saroj Kumar Chaudhary**: formal analysis and investigation. **Hazem S. Elshafie**: writing – original draft preparation, writing – review and editing. **Vincenzo De Feo**: writing – review and editing.

## Conflicts of Interest

The authors declare no conflicts of interest.

## Data Availability

The data that support the findings of this study are available from the corresponding author upon reasonable request.
